# Prophylactic antibiotics for pancreaticoduodenectomy: study protocol for a multicenter randomized controlled trial comparing tazobactam/piperacillin versus ampicillin/sulbactam

**DOI:** 10.1186/s12893-026-04092-8

**Published:** 2026-08-01

**Authors:** Takuya Mizumoto, Yoshihide Nanno, Jun Ishida, Yuki Ueda, Dongha Lee, Masayuki Akita, Riki Asakura, Hiroyuki Nakamura, Taisuke Okawa, Masaharu Yokota, Koki Hamano, Shohei Komatsu

**Affiliations:** Department of Surgery, Division of Hepato-Biliary-Pancreatic Surgery, Kobe University Graduate School of Medicine, 7-5-2 Kusunoki-Cho, Chuo-ku, Kobe, 650-0017 Japan

**Keywords:** Surgical site infection, Pancreatoduodenectomy, Antibiotics, Prophylaxis, Tazobactam/piperacillin, Ampicillin/sulbactam

## Abstract

**Background:**

Pancreaticoduodenectomy (PD) is associated with a high incidence of surgical site infection (SSI), reported in 20–40% of cases. In patients undergoing preoperative biliary drainage (PBD), the risk is further increased due to biliary contamination. Tazobactam/piperacillin (TAZ/PIPC) has been shown to reduce SSI compared with cephalosporins; however, no prospective trial has directly compared TAZ/PIPC with ampicillin/sulbactam (ABPC/SBT), which is widely used in pancreatic surgery. This study aims to evaluate the superiority of TAZ/PIPC over ABPC/SBT in preventing SSI after PD.

**Methods:**

This is a multicenter, open-label, randomized controlled trial. A total of 320 patients undergoing PD will be randomized (1:1) to receive either TAZ/PIPC or ABPC/SBT as perioperative prophylactic antibiotics. Randomization will be stratified by PBD, minimally invasive surgery (MIS), and pancreatic ductal adenocarcinoma (PDAC). Antibiotics will be administered within 60 min before incision, redosed intraoperatively every 2–4 h, and continued every 8 h until postoperative day 1. The primary endpoint is the incidence of SSI within 30 days postoperatively, defined according to CDC criteria. Secondary endpoints include postoperative complications (Clavien–Dindo ≥IIIa), clinically relevant postoperative pancreatic fistula (ISGPS grade B/C), delayed gastric emptying, postpancreatectomy hemorrhage, bile leakage, mortality, reoperation, readmission, C. difficile colitis, and detection of antimicrobial-resistant organisms.

**Discussion:**

This trial will provide the first direct comparison between TAZ/PIPC and ABPC/SBT in PD. The results are expected to contribute to optimizing perioperative antibiotic strategies while balancing SSI prevention and antimicrobial resistance.

**Trial registration:**

Japan Registry of Clinical Trials (jRCT), jRCTs051260063.

**Supplementary Information:**

The online version contains supplementary material available at https://doi.org/10.1186/s12893-026-04092-8.

## Background

Pancreaticoduodenectomy (PD) is one of the most invasive procedures in gastrointestinal surgery and is associated with a high incidence of postoperative complications, particularly surgical site infection (SSI), with reported rates ranging from 20% to 40% [1]. SSI is associated with prolonged hospital stay, increased rates of reoperation and readmission, higher healthcare costs, and delays in the initiation of adjuvant therapy, potentially affecting long-term outcomes. Therefore, prevention of SSI remains a critical issue in the perioperative management of PD. Patients undergoing preoperative biliary drainage (PBD) are at particularly high risk of SSI due to bacterial contamination of bile [2]. Accordingly, the selection of prophylactic antibiotics with adequate coverage against biliary pathogens is essential in this setting. A large randomized controlled trial comparing tazobactam/piperacillin (TAZ/PIPC), a broad-spectrum β-lactam/β-lactamase inhibitor combination, with the second-generation cephalosporin cefoxitin demonstrated a significantly lower incidence of SSI in the TAZ/PIPC group (19.8% vs. 32.8%, *p* < 0.001) [3]. Based on these findings, several international guidelines have incorporated TAZ/PIPC as a prophylactic antibiotic option for PD [4, 5]. However, substantial variation in prophylactic antibiotic practices persists across institutions. Ampicillin/sulbactam (ABPC/SBT), another β-lactam/β-lactamase inhibitor combination, has antimicrobial activity against many common biliary pathogens and is frequently used as a prophylactic antibiotic in hepato-biliary-pancreatic surgery [6]. Emerging evidence suggests that SSI after PD may involve organisms resistant to ABPC/SBT, raising concerns regarding the adequacy of its antimicrobial coverage [7]. Large-scale data regarding ABPC/SBT prophylaxis in patients undergoing PD are limited. However, retrospective data from participating institutions suggest SSI rates ranging from approximately 30% to 40%, which may be higher than those reported with TAZ/PIPC in previous studies.

While TAZ/PIPC provides broad antimicrobial coverage against pathogens associated with SSI after PD, its widespread use may contribute to the emergence of antimicrobial resistance. Therefore, it is essential to evaluate the balance between clinical efficacy and the potential risk of resistance. In addition, most previous studies evaluating prophylactic antibiotics in PD were conducted primarily in open surgery populations, whereas evidence in minimally invasive surgeries (MIS) remains limited. Given the increasing adoption of minimally invasive approaches and the potential differences in contamination patterns and postoperative infectious complications, validation of antibiotic strategies in a study including both open and minimally invasive PD is clinically relevant.

Although both TAZ/PIPC and ABPC/SBT are widely used in clinical practice and have well-established safety profiles, no prospective study has directly compared these two regimens as prophylactic antibiotics for PD. This represents an important gap in the current evidence base.

The aim of this study is to test the hypothesis that TAZ/PIPC reduces the incidence of SSI after PD compared with ABPC/SBT.

## Methods

### Study design

This multicenter, open-label, randomized, parallel-group comparative study was designed to evaluate the superiority of prophylactic TAZ/PIPC over ABPC/SBT in preventing SSI in patients undergoing PD. Potential participants include all adult patients scheduled to undergo PD during the study period who meet the eligibility criteria. Eligible patients will be randomized in a 1:1 ratio to receive perioperative prophylactic antibiotics with either TAZ/PIPC or ABPC/SBT. Randomization will be stratified according to PBD, MIS, and pancreatic ductal adenocarcinoma (PDAC).

The primary endpoint is the incidence of SSI within 30 days after surgery, defined according to the Centers for Disease Control and Prevention (CDC) criteria [8]. Secondary endpoints include major postoperative complications, clinically relevant postoperative pancreatic fistula, delayed gastric emptying, postpancreatectomy hemorrhage, bile leakage, mortality, and emergence of antimicrobial-resistant organisms. The study will be conducted at 13 institutions in Japan, including academic referral centers and affiliated regional hospitals with experience in PD. Participant recruitment will be distributed across the participating institutions according to their annual surgical volumes. Based on the expected case volumes, enrollment of 320 patients during the planned study period is considered feasible.

**Fig. 1:**
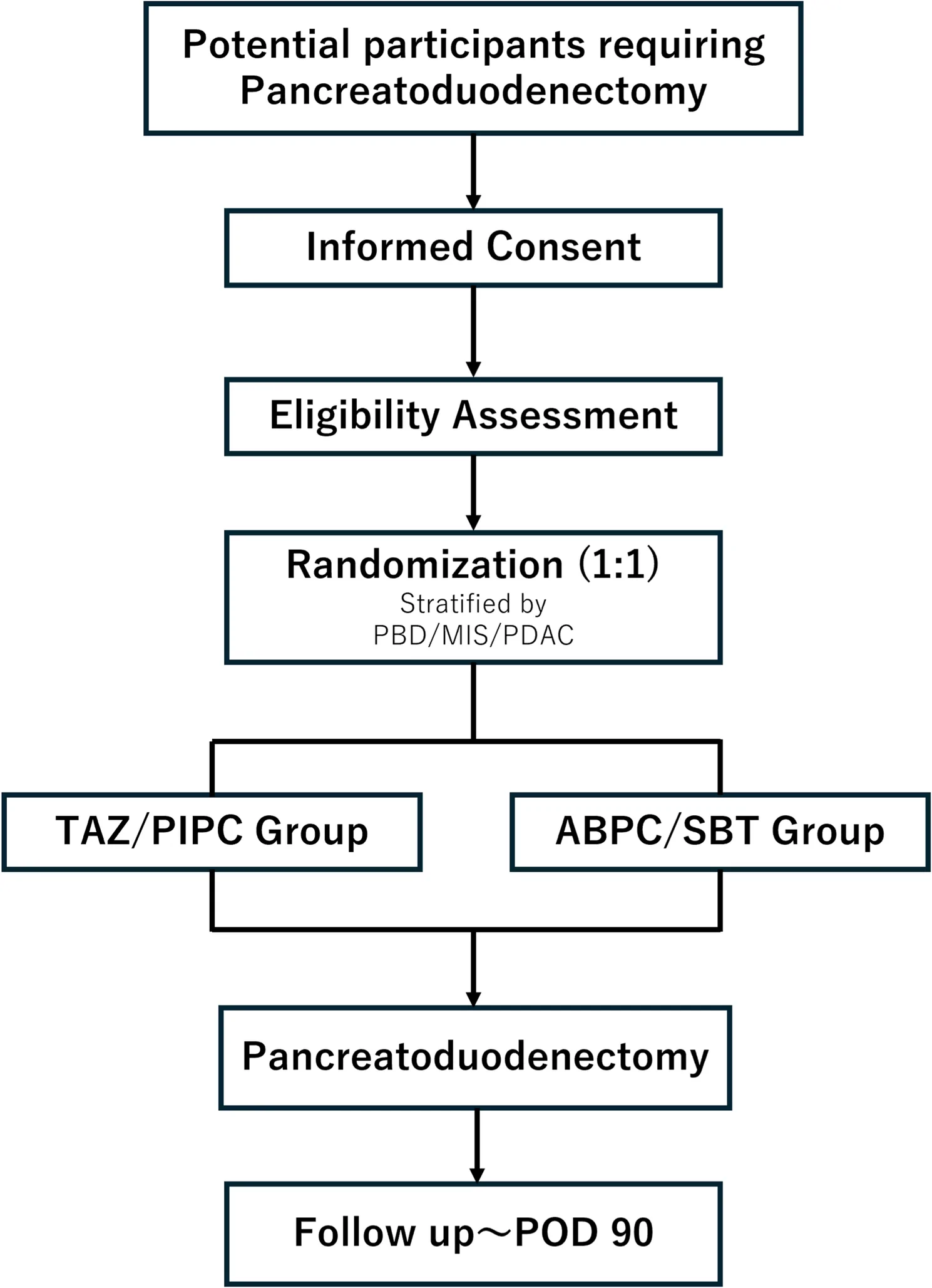
Study flow diagram. Eligible patients undergoing pancreaticoduodenectomy are randomized in a 1:1 ratio to receive perioperative prophylactic antibiotics with either TAZ/PIPC or ABPC/SBT. Participants are followed for 90 days after surgery. The primary endpoint is the occurrence of surgical site infection within 30 days after surgery

A summary of the study design is shown in Fig. 1.

This study is conducted as a specified clinical trial under the Japanese Clinical Trials Act and has been approved by the certified review board (CRB) of Kobe University (approval no. C260002). The trial has been registered in the Japan Registry of Clinical Trials (jRCT; https://jrct.mhlw.go.jp/en-top) under identifier jRCTs051260063.

### Eligibility criteria

Eligibility will be assessed at enrollment before randomization.

#### Inclusion criteria

Participants meeting all of the following criteria will be included:

1. Age 18 years or older at the time of informed consent.
2. Scheduled to undergo PD (including pylorus-preserving PD and subtotal stomach-preserving PD).
3. Written informed consent obtained after a full explanation of the study.

#### Exclusion criteria

Participants who meet at least one of the following criteria will be excluded:

1. History of hypersensitivity to TAZ/PIPC, ABPC/SBT, penicillin antibiotics, or any excipients in these agents.
2. Severe renal dysfunction (creatinine clearance < 40 mL/min).
3. Pregnancy, possible pregnancy, or breastfeeding.
4. Infectious mononucleosis.
5. Planned concomitant resection of other organs beyond the scope of standard PD (excluding vascular resection/reconstruction).
6. Participation in another interventional clinical study that may affect the outcomes of this trial.
7. Participants deemed unsuitable for participation by the investigators.

### Randomization and allocation

Eligible participants will be randomized in a 1:1 ratio using a stratified permuted block method with variable block sizes of 2 and 4. Randomization will be stratified by PBD (yes/no), scheduled for MIS (yes/no), and PDAC (yes/no). Randomization will be performed using the web-based electronic data capture system REDCap. Treatment allocation will be disclosed after enrollment and before surgery to enable appropriate perioperative antibiotic administration. Because of the nature of the intervention, blinding will not be performed for patients, surgeons, or investigators.

### Schedule of the study

Following written informed consent, eligibility criteria will be assessed and baseline data collected. Eligible participants will be randomized in a 1:1 ratio to receive either TAZ/PIPC or ABPC/SBT as perioperative prophylactic antibiotics. The allocated prophylactic antibiotic regimen will be administered according to the study protocol from induction of anesthesia until postoperative day 1. Participants will be followed during hospitalization and for 90 days after surgery to assess SSI, postoperative complications, adverse events, and other study outcomes.

The schedule of enrolment, interventions, and assessments is summarized in Fig. 1; Table 1 in accordance with the Standard Protocol Items: Recommendations for Interventional Trials (SPIRIT) statement. SSI was assessed continuously during the 30-day postoperative period and adjudicated at postoperative day 30.

**Table 1:** Schedule of enrolment, interventions, and assessments according to the SPIRIT statement

Study period	Screening	Allocation	Surgery	Postoperative period	Day 30	Day 90
Enrolment						
Informed consent	X					
Eligibility assessment	X					
Randomization		X				
Baseline characteristics	X					
Interventions						
TAZ/PIPC or ABPC/SBT administration			X	X (day1)		
Pancreaticoduodenectomy			X			
Assessments						
Surgical site infection (primary endpoint)					X	
Superficial/deep/organ-space SSI					X	
Clinically relevant POPF (ISGPS grade B/C)				X		
Delayed gastric emptying (ISGPS)				X		
Postpancreatectomy hemorrhage (ISGPS)				X		
Clavien–Dindo ≥ Grade IIIa complications						X
Adverse events			X	X	X	X
Clostridium difficile colitis				X		
Antimicrobial-resistant organisms				X		
Reoperation						X
Readmission						X
Mortality						X

### Intervention

Participants randomized to the TAZ/PIPC group will receive TAZ/PIPC 4.5 g administered within 60 min before skin incision, with intraoperative redosing every 2–4 h as appropriate, and continued every 8 h until postoperative day 1. Participants randomized to the ABPC/SBT group will receive ABPC/SBT 3.0 g according to the same schedule. No prophylactic antibiotics will be administered after postoperative day 1. Antibiotic administration initiated on or after postoperative day 2 for suspected or confirmed infection will be regarded as therapeutic treatment and will be distinguished from protocol-defined prophylactic antibiotic administration. The allocated prophylactic antibiotic regimen will be administered regardless of the results of preoperative bile culture or antimicrobial susceptibility testing. If therapeutic antimicrobial treatment is clinically indicated after surgery, antimicrobial therapy may be modified at the discretion of the treating physicians. Perioperative management other than prophylactic antibiotic administration will be performed according to institutional standards and established SSI prevention guidelines.

## Results

### Primary endpoint

The primary endpoint is the occurrence of any SSI within 30 days after surgery. SSI will be assessed according to the CDC criteria and classified as superficial incisional SSI, deep incisional SSI, or organ/space SSI. ^8^ SSI will be assessed by the treating physicians at each participating institution according to the standardized CDC criteria and the predefined diagnostic definitions specified in the study protocol to ensure consistent assessment across participating centers. Detailed definitions of SSI are provided in Supplementary Appendix 1.

### Secondary endpoints

The secondary endpoints are as follows:

#### Efficacy endpoints

1. Incidence of superficial incisional SSI, deep incisional SSI, and organ/space SSI within 30 days after surgery.
2. Incidence of postoperative complications of Clavien–Dindo grade IIIa or higher within 90 days after surgery [9].
3. Incidence of clinically relevant postoperative pancreatic fistula (POPF; ISGPS grade B/C) [10].
4. Incidence of delayed gastric emptying (DGE) of any grade according to the ISGPS criteria [11].
5. Incidence of postpancreatectomy hemorrhage (PPH; ISGPS grade B/C) [12].
6. Incidence of postoperative bile leakage (ISGLS grade B/C) [13].
7. Length of postoperative hospital stay.
8. Mortality within 90 days after surgery.
9. Reoperation within 90 days after surgery.
10. Readmission within 90 days after surgery.
11. Incidence of postoperative infectious complications, including intra-abdominal abscess, bacteremia, cholangitis, pneumonia, urinary tract infection, and catheter-related infection.

#### Safety endpoints

1. Adverse events associated with prophylactic antibiotic administration.
2. Incidence of Clostridium difficile colitis.
3. Detection of antimicrobial-resistant organisms, including extended-spectrum β-lactamase (ESBL)-producing organisms, carbapenem-resistant Enterobacterales (CRE), methicillin-resistant Staphylococcus aureus (MRSA), vancomycin-resistant Enterococcus (VRE), multidrug-resistant Gram-negative organisms, and Candida species.

#### Exploratory endpoints

1. Risk factors for clinically relevant POPF (ISGPS grade B/C) in patients undergoing open and minimally invasive PD.
2. Risk factors for SSI in patients undergoing open and minimally invasive PD.
3. Associations between microorganisms and antimicrobial susceptibility identified in preoperative bile cultures, intraoperative bile cultures, and postoperative drain cultures and the development of postoperative infectious complications.
4. Distribution of causative microorganisms in postoperative infectious complications.
5. Detection patterns of multidrug-resistant organisms in postoperative infectious complications.
6. Association between PBD and postoperative infectious complications.

### Sample size calculation

The primary hypothesis of this study is that TAZ/PIPC is superior to ABPC/SBT in reducing the incidence of SSI after PD. A previous randomized controlled trial involving 778 patients undergoing PD demonstrated a significantly lower incidence of SSI in patients receiving TAZ/PIPC than in those receiving cefoxitin (19.8% vs. 32.8%).^3^ Furthermore, a recent systematic review including 12,469 patients reported an SSI incidence of 20.1% among patients receiving broad-spectrum prophylactic antibiotics, including TAZ/PIPC [14]. Large-scale data regarding ABPC/SBT in this setting are limited. The assumed SSI rate in the ABPC/SBT group was based on retrospective data from two participating institutions. At Kobe University Hospital, the incidence of SSI was 38.8% among 80 consecutive patients who received ABPC/SBT as prophylactic antibiotics in 2024, of whom 35.0% underwent PBD. At Fujita Health University, the SSI incidence was 32.8% among 116 patients treated with ABPC/SBT between 2022 and 2023, with a PBD rate of 41.3%. Based on these findings, the SSI incidence was assumed to be 20% in the TAZ/PIPC group and 35% in the ABPC/SBT group. Using Pearson’s chi-square test with a two-sided significance level of 0.05 and a statistical power of 80%, 138 patients per group are required to detect this difference. Assuming a dropout rate of approximately 15%, the target sample size was set at 160 patients per group, resulting in a total sample size of 320 patients.

### Data management

Study data will be collected and managed using the REDCap electronic data capture system. Investigators at each participating center will enter study data into electronic case report forms (eCRFs) using individual user accounts with password-protected access. Data quality will be ensured through predefined validation rules, regular data monitoring, and query resolution procedures. Investigators will review entered data and electronically certify the accuracy and completeness of the eCRFs. Source documents will include informed consent forms, medical records, nursing records, laboratory test results, imaging findings, operative records, and other documents required under applicable regulations. Data recorded in the electronic medical record system will be considered source data. All study data will be handled in accordance with applicable regulations and institutional policies regarding confidentiality and data protection. Access to the study database will be restricted to authorized study personnel.

### Data analysis

#### Analysis populations

Three analysis populations will be defined as follows:

##### Full analysis set

The Full Analysis Set (FAS) will include all randomized participants who receive the allocated study drug and undergo PD. Participants will be analyzed according to the treatment group to which they were originally assigned, representing a modified intention-to-treat (mITT) population. Participants meeting any of the following criteria will be excluded from the FAS:

1. No administration of the allocated study drug.
2. Withdrawal of consent with refusal of data use.
3. Failure to undergo PD because of intraoperative findings or changes in the surgical procedure.

##### Per protocol set

The Per Protocol Set (PPS) will consist of participants in the FAS who do not have major protocol violations. The PPS analysis will be performed as a sensitivity analysis to assess the robustness of the primary efficacy results.

Major protocol deviations include:

1. Violation of inclusion criteria.
2. Violation of exclusion criteria.
3. Protocol deviations that may substantially affect efficacy assessment.

##### Safety analysis set

The Safety Analysis Set (SAS) will include all participants who receive at least one dose of the allocated study drug. Safety analyses will be performed using the SAS.

### Handling of cases

Participants may withdraw from the study at any time without providing a reason and without any effect on their subsequent medical care.

Study participation will be discontinued if any of the following conditions are met:

1. Withdrawal of consent by the participant.
2. Determination after enrollment that the participant did not meet the eligibility criteria.
3. Cancellation of PD or conversion to another surgical procedure based on preoperative or intraoperative findings.
4. Discontinuation of the allocated study drug because of worsening renal function, allergic reactions, or other safety concerns.
5. Inability to continue study participation because of deterioration of concomitant medical conditions.
6. Occurrence of adverse events that preclude further participation in the study.
7. Premature termination of the study.
8. Any other reason for which the investigators consider discontinuation to be in the participant’s best interest.

Whenever possible, participants who discontinue study treatment will continue to be followed for outcome assessment according to the study protocol.

### Handling of data

Study data will be collected using eCRFs and managed through a secure electronic data capture system. Data queries, discrepancies, and uncertainties identified during data review will be resolved through discussion between the study coordinator and the lead biostatistician according to predefined procedures. The handling of missing, excluded, or anomalous data will be determined prior to database lock and documented in the statistical analysis plan. Any issues regarding case eligibility or data interpretation that arise during the study will be reviewed jointly by the study coordinator and the lead biostatistician. Participants who discontinue study participation will be included in the analysis using all available data collected up to the time of discontinuation unless consent for data use is withdrawn. In cases of withdrawal of consent for data use, the participant’s data will be excluded to the extent permitted by applicable regulations. All statistical analyses will be performed after completion of study treatment and database lock. Efficacy analyses will be conducted primarily in the Full Analysis Set (FAS), with supportive analyses performed in the Per Protocol Set (PPS). Safety analyses will be conducted in the Safety Analysis Set (SAS).

### Summarization of subject baseline characteristics

Baseline characteristics will be summarized for each analysis population by treatment group. Categorical variables will be presented as frequencies and percentages. Continuous variables will be summarized using descriptive statistics, including the number of observations, mean, standard deviation, minimum, first quartile, median, third quartile, and maximum. Baseline characteristics will be described to assess the comparability of the treatment groups; no formal statistical testing of baseline variables is planned.

### Primary endpoint analysis

The primary endpoint is the occurrence of SSI within 30 days after PD according to the CDC criteria. The incidence of SSI will be calculated for each treatment group and compared using Pearson’s chi-square test with a two-sided significance level of 0.05. Treatment effects will be expressed as the risk difference and risk ratio with corresponding 95% confidence intervals. Deaths within 30 days attributable to infectious complications will be considered SSI events. Deaths unrelated to infection within 30 days after surgery will be treated as non-events for the primary endpoint analysis.

### Secondary endpoint analysis

Secondary endpoint analyses will be considered exploratory. P values will be reported as descriptive measures, and no adjustment for multiplicity will be performed. Binary outcomes, including superficial incisional SSI, deep incisional SSI, organ/space SSI, Clavien–Dindo grade IIIa or higher complications, clinically relevant POPF (ISGPS grade B/C), DGE, PPH, 90-day mortality, reoperation, readmission, and postoperative infectious complications, will be summarized as frequencies and percentages and compared using Pearson’s chi-square test or Fisher’s exact test, as appropriate. Risk differences and corresponding 95% confidence intervals will also be calculated. Postoperative length of hospital stay will be summarized using descriptive statistics. The distribution of postoperative length of stay will be assessed prior to analysis. If the assumption of normality is satisfied, between-group comparisons will be performed using the two-sample *t*-test; otherwise, the Mann–Whitney *U* test will be used. Prespecified subgroup analyses will be performed according to the presence or absence of PBD and MIS. The primary and secondary endpoints will be analyzed within each subgroup using the same analytical approach as described above. These subgroup analyses are exploratory in nature, and no formal treatment-by-subgroup interaction tests are planned.

### Safety endpoint analysis

Safety analyses will be performed in the Safety Analysis Set (SAS). Safety outcomes include adverse events related to prophylactic antibiotic administration, Clostridioides difficile infection, and the detection of antimicrobial-resistant organisms, including extended-spectrum β-lactamase-producing organisms (ESBL), carbapenem-resistant Enterobacterales (CRE), methicillin-resistant Staphylococcus aureus (MRSA), vancomycin-resistant Enterococcus (VRE), and multidrug-resistant Gram-negative organisms. The incidence and proportion of each safety outcome will be summarized by treatment group using descriptive statistics. No formal hypothesis testing is planned for safety outcomes.

### Exploratory analyses

Exploratory analyses will be performed to identify risk factors for clinically relevant POPF (ISGPS grade B/C) and postoperative SSI after open and minimally invasive PD. Patient characteristics, operative factors, surgical approach, and PBD status will be evaluated. Independent risk factors for clinically relevant POPF and SSI will be evaluated using multivariable logistic regression analysis. Candidate covariates will be selected based on clinical relevance and the results of univariable analyses. Associations between microorgaindependentified from preoperative bile cultures, intraoperative bile cultures, and postoperative drain cultures and the development of postoperative infectious complications will also be investigated. Microbial species distribution, antimicrobial susceptibility patterns, and the detection of multidrug-resistant organisms will be summarized descriptively. The association between PBD and postoperative infectious complications will be assessed using comparative and multivariable analyses.

### Data and safe monitoring

A monitoring process will be implemented to ensure study quality, protocol adherence, and participant safety. Periodic internal monitoring will be conducted by the study coordinating team in accordance with the Clinical Trials Act in Japan and the study protocol. The monitoring process will include review of informed consent procedures and documentation, participant enrollment and retention, protocol compliance, safety data, data completeness and consistency, and overall study progress. Data queries and protocol deviations identified during monitoring will be reviewed by the study coordinator and investigators and resolved according to predefined procedures. Any significant findings related to participant safety or study conduct will be reported to the certified review board as required. An independent data and safety monitoring board has not been established because this investigator-initiated study involves perioperative use of approved antibiotics within standard clinical practice and is considered to have a relatively low risk profile.

### Patients and public involvement

This research was not conducted with the involvement of patients or members of the public in its design, conduct, reporting, or dissemination.

### Confidentiality

To ensure participant confidentiality, all data will be coded using unique identification numbers. Identifiable personal information will be stored separately from study data in locked cabinets located in restricted-access areas. Electronic data will be stored on password-protected computers and secure institutional servers. Study-related documents and data will be retained for at least five years following study completion. No identifiable information will be shared outside the study without explicit written consent from the participants.

### Ethics and dissemination

This clinical trial has been approved by the CRB of Kobe University Hospital. The study protocol is registered with the jRCT, and it will be conducted in full compliance with the Clinical Trials Act of Japan, the Declaration of Helsinki (2013 revision), and all applicable regulations and institutional guidelines. Prior to enrollment, written informed consent will be obtained from all participants after a thorough explanation of the study’s objectives, procedures, risks, and potential benefits. Participation is voluntary, and patients may withdraw from the study at any time without penalty or loss of access to medical care. The safety of participants is of paramount importance. In the event of an adverse event, appropriate clinical management will be provided. Compensation for study-related injuries will be offered in accordance with institutional policies and the Clinical Trials Act. The study’s results, encompassing positive, negative, or inconclusive findings, will be disseminated through publication in peer-reviewed journals and presentations at academic conferences, while ensuring no personally identifiable information is disclosed. No personally identifiable information will be disclosed in any publications or presentations.

## Discussion

Despite continuous improvements in surgical techniques and perioperative care, postoperative infectious complications remain a major source of morbidity after PD. SSI is among the most frequent complications after PD and continues to represent an important target for quality improvement in pancreatic surgery [1]. Therefore, optimization of perioperative antibiotic prophylaxis remains a clinically important issue.

The choice of prophylactic antibiotics in PD is still controversial. Previous studies demonstrated that broad-spectrum antibiotic regimens, including TAZ/PIPC, may reduce SSI compared with conventional cephalosporin-based prophylaxis, and several international guidelines have incorporated TAZ/PIPC as a prophylactic option for PD [4, 5]. However, substantial variation in clinical practice persists, and ABPC/SBT remains widely used in hepato-biliary-pancreatic surgery because of its activity against common biliary pathogens [6].

To date, no prospective randomized study has directly compared TAZ/PIPC and ABPC/SBT in patients undergoing PD. Recently, the PERIoperative antibiotic prophylaxis in PANCreatic surgery (PeriPANC) study was initiated in Germany to prospectively compare TAZ/PIPC and ABPC/SBT in pancreatic surgery [4]. However, the study is observational in nature and includes various types of pancreatic resections. Consequently, robust randomized evidence focusing specifically on patients undergoing PD is still lacking. Moreover, most previous studies evaluating prophylactic antibiotic strategies in pancreatic surgery have been conducted predominantly in open surgery populations. Given the increasing adoption of minimally invasive PD worldwide, evidence regarding optimal antibiotic prophylaxis in both open and minimally invasive procedures is increasingly needed. Therefore, the present multicenter randomized trial was designed to directly compare these two commonly used antibiotic regimens under contemporary clinical practice conditions.

Another unresolved issue in PD is the optimal duration of prophylactic antibiotic administration. A recent systematic review suggested that prolonged antibiotic prophylaxis may reduce organ/space SSI in patients undergoing PBD, whereas other observational studies have demonstrated no clear benefit of extended antibiotic administration [15–17]. Thus, the optimal duration of prophylaxis remains uncertain and warrants further investigation. The present trial was specifically designed to evaluate the choice of prophylactic antibiotic agents under a standardized administration period rather than the duration of antibiotic therapy.

The present study may also contribute to the ongoing discussion regarding the balance between antimicrobial efficacy and antimicrobial stewardship. Although TAZ/PIPC provides broader antimicrobial coverage against pathogens associated with postoperative infection, routine use of broad-spectrum antibiotics may increase selective pressure for antimicrobial resistance. By evaluating not only SSI but also Clostridioides difficile infection and the emergence of antimicrobial-resistant organisms, this trial is expected to provide clinically meaningful information regarding both effectiveness and safety. The findings of this study may have direct implications for clinical practice. If TAZ/PIPC is shown to significantly reduce SSI compared with ABPC/SBT without substantially increasing antimicrobial-resistant organisms or Clostridium difficile infection, broader adoption of TAZ/PIPC may be justified in patients undergoing PD. Conversely, if no clinically meaningful benefit is demonstrated, the routine use of broader-spectrum antibiotics may be difficult to justify from an antimicrobial stewardship perspective. Thus, the findings of this trial may help establish evidence-based recommendations for prophylactic antibiotic selection after PD while balancing antimicrobial efficacy against the risk of promoting antimicrobial resistance.

Several limitations should be acknowledged. First, the study employs an open-label design, which may introduce performance bias. However, the primary endpoint is based on standardized CDC criteria and predefined diagnostic definitions, thereby minimizing assessment bias. Second, perioperative management other than prophylactic antibiotic administration is not completely standardized across participating institutions. Nevertheless, although complete standardization of perioperative management cannot be guaranteed, participating centers employ broadly similar perioperative care pathways and follow a common study protocol, thereby supporting the clinical relevance and generalizability of the findings. Third, the trial was not designed to determine the optimal duration of prophylactic antibiotic administration, and further studies are warranted to establish patient-tailored strategies according to individual risk factors such as PBD and microbiological findings. Finally, the study is not powered for subgroup analyses according to PBD status or surgical approach, and these analyses should therefore be considered exploratory.

## Supplementary Information

Supplementary Material 1.

## Data Availability

No datasets were generated or analysed during the current study.

## Abbreviations

ABPC/SBT: Ampicillin/sulbactam
CDC: Centers for Disease Control and Prevention
CDI: Clostridioides difficile infection
CR-POPF: Clinically relevant postoperative pancreatic fistula
CRE: Carbapenem-resistant Enterobacterales
DGE: Delayed gastric emptying
ESBL: Extended-spectrum β-lactamase
FAS: Full Analysis Set
ISGPS: International Study Group of Pancreatic Surgery
MIS: Minimally invasive surgery
MRSA: Methicillin-resistant Staphylococcus aureus
PBD: Preoperative biliary drainage
PD: Pancreaticoduodenectomy
PDAC: Pancreatic ductal adenocarcinoma
POPF: Post-operative pancreatic fistula
PPH: Postpancreatectomy hemorrhage
PPS: Per Protocol Set
SAS: Safety Analysis Set
SAP: Statistical Analysis Plan
SSI: Surgical site infection
TAZ/PIPC: Tazobactam/piperacillin
VRE: Vancomycin-resistant Enterococcus
