# Supplementary material for: Prophylactic antibiotics for pancreaticoduodenectomy: study protocol for a multicenter randomized controlled trial comparing tazobactam/piperacillin versus ampicillin/sulbactam
Source: BMC Surg. 2026 Aug 1;26:726. doi: 10.1186/s12893-026-04092-8 (PMC13717995; doi:10.1186/s12893-026-04092-8)
Supplement: Supplementary file 1 [file 12893_2026_4092_MOESM1_ESM.docx]

# Supplementary Appendix 1. Definitions of Surgical Site Infection

Surgical site infection (SSI) will be defined according to the Centers for Disease Control and Prevention (CDC) criteria. SSI occurring within 30 days after pancreaticoduodenectomy will be classified into the following categories.

## 1. Superficial incisional SSI

Superficial incisional SSI involves only the skin and subcutaneous tissue of the incision and must meet the CDC criteria for superficial incisional infection.

At least one of the following conditions must be present:

- Purulent drainage from the superficial incision.
- Organisms identified from an aseptically obtained specimen of fluid or tissue from the superficial incision.
- The superficial incision is deliberately opened by a surgeon or physician and the patient has signs or symptoms of infection, such as pain, tenderness, localized swelling, erythema, or heat.
- Diagnosis of superficial incisional SSI by the surgeon or attending physician.

## 2. Deep incisional SSI

Deep incisional SSI involves the deep soft tissues of the incision, including fascial and muscle layers, and must meet the CDC criteria for deep incisional infection.

At least one of the following conditions must be present:

- Purulent drainage from the deep incision.
- A deep incision that spontaneously dehisces or is deliberately opened by a surgeon or physician when the patient has signs or symptoms of infection, such as fever, localized pain, or tenderness.
- Evidence of deep incisional infection identified by direct examination, reoperation, histopathological examination, or radiological imaging.
- Diagnosis of deep incisional SSI by the surgeon or attending physician.

## 3. Organ/space SSI

Organ/space SSI involves any part of the body that is opened or manipulated during the surgical procedure, excluding the incision, fascia, and muscle layers, and must meet the CDC criteria for organ/space infection.

At least one of the following conditions must be present:

- Purulent drainage from a drain placed into the organ or space.
- Organisms identified from an aseptically obtained specimen of fluid or tissue from the organ or space.
- Evidence of infection involving the organ or space identified by direct examination, reoperation, histopathological examination, or radiological imaging.
- Diagnosis of organ/space SSI by the surgeon or attending physician.

For the purpose of the present study, the primary endpoint will be defined as the occurrence of any SSI, including superficial incisional SSI, deep incisional SSI, or organ/space SSI, within 30 days after pancreaticoduodenectomy.
